# Effectiveness of lever sign test for diagnosing anterior cruciate ligament rupture

**DOI:** 10.12669/pjms.38.4.4993

**Published:** 2022

**Authors:** Naveed Ali Shair, Umair Abubakar Siddiq, Abdullah Tariq, Muhammad Khalid

**Affiliations:** 1 Naveed Ali Shair, FCPS (Ortho), Department of Orthopedic Surgery, Lahore General Hospital, Lahore, Pakistan; 2 Umair Abubakar Siddiq, FCPS (Ortho) Department of Orthopedic Surgery, Central Park Medical College Lahore, Pakistan; 3 Abdullah Tariq, FCPS (Ortho) Department of Orthopedic Surgery, DHQ Hopsital, Nanakana Sahab, Lahore, Pakistan; 4 Muhammad Khalid, FCPS (Ortho), Department of Orthopedic Surgery, Lahore General Hospital, Lahore, Pakistan

**Keywords:** Anterior cruciate ligament, Lever sign test, Magnetic resonance imaging

## Abstract

**Objective:**

To evaluate the effectiveness of the Lever Sign test (LST) for diagnosing anterior cruciate ligament (ACL) ruptures.

**Methods:**

This prospective trial was conducted from January to December 2020. A total of 73 patients, aged 18 to 65 years, presenting with chief complaint as acute knee pain rated < 7/10 on a verbal numerical rating scale, having a minimum 20 to 120° range of motion and undergoing magnetic resonance imaging (MRI) were enrolled. Detailed history, physical examination and standard radiographic evaluation were done in all subjects. For the assessment of the integrity of the ACL, the anterior drawer, Lachman, pivot-shift and LST were performed on each symptomatic knee by a senior orthopedic consultant with a minimum five years post-fellowship experience. Sensitivity, specificity, positive predictive value (PPV), negative predictive value (NPV) and accuracy of the LST were recorded with respect to standard reference MRI findings.

**Results:**

Out of a total of 73 patients, there were 49 (67.1%) males. Mean age was noted to be 34.5±8.1 years. Area of residence was rural among 42 (57.5%) patients. Mean time since injury was noted to be 11.2±4.6 months. The MRI findings showed ACL intact among 31 (42.4%) patients while it showed ACL torn among 42 (57.5%) patients. The LST showed positive findings for ACL rupture in 39 (53.5%) patients while it was negative in remaining 34 (46.5%). The sensitivity, specificity, PPV, NPV and accuracy of LST with respect to standard reference finding (MRI) was found to be 86%, 90%, 92%, 82% and 88% respectively.

**Conclusion:**

The LST was found to have good specificity, sensitivity, PPV, NPV and accuracy to detect the status of the ACL. The LST is easy to perform can be used along with other standard assessment techniques to further increase the validation of the status of the ACL diagnosis.

## INTRODUCTION

The anterior cruciate ligament (ACL) is the most frequently involved knee injuries.^1^ ACL tears are calculated to be responsible for around 20% of all knee injuries while studies from USA estimated about 120,000 ACL injuries annually.^2^,^3^

Arthroscopic visualization is known to be the “Gold Standard” used for diagnosing ACL rupture.^4^ Magnetic resonance imaging (MRI) is considered to be a valid yet non-invasive tool for the diagnosis of the ACL ruptures with specificity and sensitivity between 94-98%.^5^ The Lachman test, the anterior Drawer test and pivot shift test are the most frequently adopted physical examinations for diagnosing ACL ruptures. All these tests are known to produce good specificity and sensitivity yet these have their own limitations like the influence of the individual guarding because of pain linked with rapidly translating or twisting a potential injury whereas it is also known that partial tears are not easy to diagnose.^6^ Even these manual methods have some limitations, yet, these have some advantages over MRI as these are totally non-invasive and easily performed while these are also inexpensive contrary to MRI.^7^

The Lever Sign Test (LST) was introduced as a new physical test and has been in use since its introduction.^7^ Some researchers have shown LST to be better or equal to other manual tests while some others have shown it to be below par in comparison to other available manual options.^8^,^9^ No data about the validity of LST exists in Pakistan so the present research was aimed at evaluating effectiveness of LST for diagnosing ACL ruptures.

## METHODS

This prospective trial was conducted at “The Department of Orthopedic Surgery, Lahore General Hospital, Lahore”, from January to December 2020. Approval from “Institutional Ethical Committee” was taken for this study (Ref#603/20, dated: 02-11-2020). Written consent was sought from all study participants.

A total of 73 patients, aged 18 to 65 years, presenting with chief complaint as acute knee pain rated < 7/10 on a verbal numerical rating scale, having a minimum 20 to 120° range of motion and undergoing MRI were enrolled. All cases with suspected fractures as per “Ottawa Knee Rules”, or having any previous knee joint arthroplasty, suspected posterior cruciate ligament involvement, past knee operations in the last six months or those who were having any major underlying non-mechanical pathological condition or systemic illnesses were not included.

Detailed history, physical examination and standard radiographic evaluation were done in all subjects. For the assessment of the integrity of the ACL, the anterior drawer, Lachman, pivot-shift and LSTs were performed on each symptomatic knee by a senior orthopedic consultant with a minimum five years post-fellowship experience. Orthopedic consultant performing the tests was blinded by the radiographic and MRI findings. The anterior drawer, Lachman and pivot-shift testes were done as described by Mulligan EP et al^10^ while LST was performed as detailed by Lelli A et al.^8^ All these four tests were done as dichotomous tests.

A special proforma was designed to record all relevant study data. For data analysis, SPSS version 26.0 was used. Gender and side involved were represented as frequency and percentages. Age (years), duration of injury (days), thigh circumference (cm) and calf circumference (cm) were represented as mean and standard deviation. Sensitivity, specificity, positive predictive value (PPV), negative predictive value (NPV) and accuracy were recorded for all the 4 types of examination maneuvers for all study cases.

## RESULTS

Out of a total of 73 patients, there were 49 (67.1%) male. Mean age was noted to be 34.5±8.1 years while 53 (72.6%) patients were above 30 years of age. Area of residence was rural among 42 (57.5%) patients. Mean time since injury was noted to be 11.2±4.6 months. Most of the cases, 40 (54.8%) had involvement of left knee. Characteristics of all cases involved in the present study are shown in Table-I.

**Table-I:** Characteristics of Study Participants (n=73).

Characteristics		No. (%) / Mean±SD
Gender	Male	49 (67.1%)
	Female	24 (32.9%)
Age (years)		34.5±8.1
Area of Residence	Rural	42 (57.5%)
	Urban	31 (42.5%)
Time Since Injury	Acute Phase (<3 weeks)	16 (21.9%)
	Sub-acute Phase (3-11 weeks)	28 (38.4%)
	Chronic Phase (≥12 weeks)	29 (39.7%)
Side Involved	Left	40 (54.8%)
	Right	33 (45.2%)
Thigh Circumference (cm)		45.8±6.1
Calf Circumference (cm)		37.4±4.2

The MRI findings showed ACL intact among 31 (42.4%) patients while it showed ACL torn among 42 (57.5%) patients. The LST showed positive findings for ACL rupture in 39 (53.5%) patients while it was negative in remaining 34 (46.5%). Sensitivity, specificity, PPV, NPV and Accuracy of LST with respect to standard reference finding (MRI) and those were found to be 86%, 90%, 92%, 82% and 88% respectively. Table-II

**Table-II:** Comparison to the Findings of Lever Sign Test in Comparison to Reference Standard MRI Findings among Study Participants (n=73).

Lever Sign Test	MRI Findings	

	Positive	Negative
Positive	36 (A)	3 (C)
Negative	6 (B)	28 (D)
Sensitivity=A/(A+B)	86%	
Specificity=D/(C+D)	90%	
Positive Predictive Value=A/(A+C)	92%	
Negative Predictive Value=D/(B+D)	82%	
Accuracy=A+D/(A+B+C+D)	88%	

Table-III is showing comparison of sensitivity, specificity, PPV, NPV and accuracy among Lever Sign and other tests performed among all study cases.

**Table-III:** Parameters of All Tests Performed versus MRI as Reference Standard (n=73).

Test	Sensitivity (%)	Specificity (%)	PPV (%)	NPV (%)	Accuracy (%)
Lever Sign	86	90	92	82	88
Anterior Drawer	88	90	93	85	89
Pivot Shift	81	84	87	76	82
Lachman	93	93	95	91	93

## DISCUSSION

In the present study, MRI was considered to be the reference standard for comparing LST and various other kinds of manual maneuver/tests for their sensitivity, specificity, PPV, NPV and accuracy. In the past, the Lachman test has been found to be the most accurate and reliable manual test used for the diagnosis ACL ruptures while pivot-shift is considered to have the least amount of sensitivity among all the most commonly used manual tests for diagnosing ACL rupture.^11^,^12^

In this study, the LST was found to have sensitivity, specificity, PPV, NPV and accuracy as 86%, 90%, 92%, 82% and 88% respectively. There are very few studies conducted to test The LST in identifying ACL tears with respect to standard references like MRI or arthroscopy but some researchers in the past have presented the LST to have comparatively better sensitivities in comparison to its contemporary manual tests among acute and chronic ACL ruptures. However, we found the LST to have a better sensitivity than pivot-shift test (86% vs. 81%) while sensitivity was comparable to anterior drawer and Lachman tests. Jarbo KA et al from USA evaluating accuracy of the LST for diagnosing ACL injuries employed different levels of testers like undergraduates, orthopedic residents and orthopedic fellows.^13^ The authors found overall sensitivity, specificity, PPV, NPV and accuracy of the LST to be 63%, 90%, 87%, 71% and 77%. The sensitivity, NPV and accuracy of the LST found by Jarbo KA et al is low that was found in the present study which could be due to the reasons that tester in the present study was an experienced orthopedic consultant (post-fellowship experience of more than five years) so this could be the reason that higher rates of sensitivity and overall accuracy were found in the present study. Difference in ours and Jarbo KA et al. studies also demonstrate that there is a possible linkage between expertise of the examiner and outcome parameters of manual test performed among cases with possible ACL tears.^13^

Lelli A et al from Italy noted LST to diagnose 100% patients with complete ACL tears of both acute and chronic types while anterior drawer, the Lachman and pivot-shift tests were found to have sensitivities of 29%, 42% and 11% respectively.^8^ Thapa SS et al. and coworkers analyzed 80 cases with knee concerns following an injury and found sensitivities of the LST, anterior drawer, Lachman and pivot-shift tests to be 86%, 80%, 91% and 51% which is close to what was found by us in the present study.^9^ Deveci et al analyzing 117 cases with confirmed diagnosis of ACL by arthroscopy compared pre-anesthesia and post-anesthesia findings of all four physical examinations. The authors noted the LST to have very good and comparable sensitivity, specificity, PPV, NPV and accuracy and concluded that the LST is an easy to apply clinical test which has higher sensitivity rates when compared to most applied the Lachman test.^14^

Although, the LST seems to be easy to apply test yielding good diagnostic validity but there seem to be few limitations as there are no validated biomechanical elucidation about the pathomechanics of the LST.^15^ Not much research has been done so far on the LST comparing this in different settings among different set patients contrary to the contemporary tests like anterior drawer, Lachman and pivot-shift tests which have large amount of studies available where these classical tests have been well studied and debated. We also don’t know much about the validity of the LST with respect to differentiation in partial or complete ACL ruptures. In strength of the LST, it is easy to learn and perform. As there is not much rapid motion of the affected knee, additional pain is minimized during this test while some researchers have also reported that the test results for the LST are not affected even if the patient is awake or under anesthesia.^13^

### Limitations of the study:

Examiners were only blinded to the MRI diagnosis but not to the affected leg or side of the injury. We were unable to compare LST findings between the injured and the uninjured legs. We did not compare arthroscopic findings about ACL rupture with LST which would have further provided validity of this test. Further research should be done to further validate the findings of the LST in comparison to other existing tests. More research is also needed to find out evaluation of the LST among different levels of examiners and clinical settings.

## CONCLUSION

The Lever Sign Test was found to have good specificity, sensitivity, PPV, NPV and accuracy to detect the status of the ACL. The Lever Sign test is easy to perform can be used along with other standard assessment techniques to further increase the validation of the status of the ACL diagnosis.

### Authors Contribution:

**NAS:** Conceived, Responsible for data’s integrity and authenticity, data collection.

**UAS:** Data Analysis, Drafting.

**AT:** Literature Review, Discussion.

**MK:** Formatting, Data Interpretation.
